# Tofacitinib combined with fire needle therapy for refractory cutaneous Rosai-Dorfman disease: a first case report and literature review

**DOI:** 10.3389/fmed.2026.1771685

**Published:** 2026-04-09

**Authors:** Yuwen Zhang, Peiyao Zheng, Bin Hu, Liuqing Chen

**Affiliations:** 1 Hubei University of Chinese Medicine, Wuhan, China; 2 Wuhan Hospital of Integrated Traditional Chinese and Western Medicine, Affiliated to Hubei University of Chinese Medicine, Wuhan, China; 3 Department of Dermatology, Wuhan First Hospital, Hubei University of Chinese Medicine, Wuhan, China

**Keywords:** cutaneous Rosai-Dorfman disease, fire needle therapy, integrated traditional Chinese and Western medicine treatment, Rosai-Dorfman disease, tofacitinib

## Abstract

A 55-year-old female patient exhibited erythematous nodular plaques on the right side of her face for more than 1 year. The dermatological examination identified mildly infiltrated erythematous patches, papules, and nodules varying in size from a wide bean to a coin on the right side of her face and nasal alae. Several nodules exhibited a yellow hue and coalesced, while the erythema presented a mildly annular configuration. The patient was diagnosed with sinus histiocytosis (Rosai-Dorfman disease) based on dermoscopy, reflectance confocal microscopy, and histopathological examination. The patient had previously had treatment at another hospital with thalidomide, methotrexate, hydroxychloroquine, and localized betamethasone injections, yielding disappointing outcomes. Subsequently, they pursued medical care at our hospital. Due to the inadequate response to the initial immunosuppressant treatment (thalidomide and methotrexate) over 2 weeks, fire needling was incorporated for an additional 2 weeks, leading to a modest improvement in the skin lesions. These treatments were then discontinued and replaced with oral tofacitinib monotherapy (5 mg twice daily), which resulted in marked regression of the skin lesions. Over a 4-month follow-up period, the skin lesions demonstrated significant flattening and fading, with no notable adverse effects observed. This case is the first report of the JAK inhibitor tofacitinib in the treatment of cutaneous Rosai-Dorfman disease, in conjunction with local fire needling intervention, providing a novel integrated therapeutic strategy combining traditional Chinese and Western medicine for refractory cases.

## 1 Introduction

Rosai-Dorfman disease (RDD), also known as sinus histiocytosis with massive lymphadenopathy, is a rare non-Langerhans cell histiocytosis of unknown etiology. The condition is characterized by the uncontrolled growth and spread of a type of white blood cell called histiocytes within the lymph nodes and surrounding tissues (1). The classic presentation of the condition is characterized by significant, painless swelling of the lymph nodes in both the sides of the neck. This is often accompanied by systemic manifestations, such as fever, night sweats, weight loss, and laboratory abnormalities including leukocytosis, an elevated erythrocyte sedimentation rate, and polyclonal hypergammaglobulinemia. Approximately 10% of cases may solely affect the skin, referred to as cutaneous Rosai-Dorfman disease (CRDD) (2). At present, there is no established therapy protocol. For patients exhibiting pronounced or refractory symptoms, surgical intervention, glucocorticoids, or immunosuppressants are frequently employed; nevertheless, success is variable across individuals, and some patients may be unable to endure the adverse reactions (3).

Recent investigations indicate that the pathogenic process of RDD is characterized by substantial immune cell infiltration and the secretion of inflammatory cytokines, implying that immune modulation may serve as a viable therapeutic target (4, 5). The Janus kinase-signal transducer and activator of transcription (JAK-STAT) signaling pathway is fundamental in controlling the function of many immune cells, including macrophages and tissue cells, as well as the synthesis of inflammatory mediators (such as IL-6 and IFN-γ) (6). Therefore, we hypothesized that tofacitinib, an oral JAK1/3 inhibitor with proven efficacy in several immune-mediated diseases (7–9), might modulate the immune microenvironment in RDD.

Tofacitinib, an oral Janus kinase (JAK) inhibitor, modulates the immune response influenced by various cytokines by obstructing the JAK-STAT signaling pathway, demonstrating potential in treating refractory inflammatory and autoimmune dermatological conditions in recent years (10). Fire needle therapy is a classic external therapeutic modality in Chinese medicine that integrates acupuncture with heat, promoting local circulation and resolving nodules. It is extensively utilized in contemporary dermatology for nodular and proliferative cutaneous lesions (11, 12). Given the immunological-inflammatory pathological characteristics of RDD, we hypothesize that employing tofacitinib for systemic immune modulation alongside fire needle for localized minimally invasive induction of immune microenvironment remodeling may yield a synergistic benefit. Currently, there are no documented instances of JAK inhibitors being utilized in conjunction with fire needle therapy for RDD, either domestically or internationally. This paper reports the first case of refractory facial CRDD that was treated using a combination of tofacitinib and fire needle therapy. While the patient achieved effective treatment with tofacitinib, fire needle therapy primarily served as an adjunctive treatment. Additionally, we conducted an extensive review of relevant domestic and international literature, summarizing current common treatment regimens for CRDD and our partial clinical experience with JAK inhibitor therapy. The aim is to provide novel, integrated Chinese and Western medicine diagnostic and therapeutic approaches for clinical practice.

## 2 Case presentation

**FIGURE 1:**
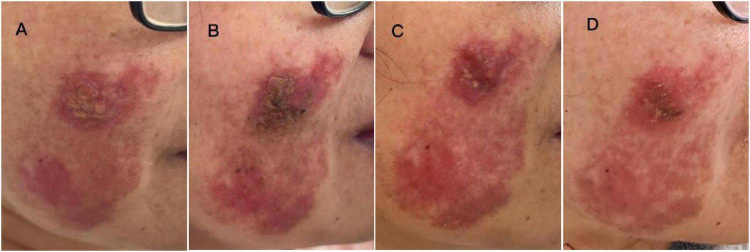
**(A)** Shows the morphology of the skin lesions at the initial visit. **(B)** Shows the skin lesions 2 weeks after combined therapy with thalidomide, methotrexate, and fire needling. **(C)** Shows the skin lesions 2 months after tofacitinib treatment. **(D)** Shows the skin lesions 4 months after tofacitinib treatment.

The patient, a 55-year-old female, presented with a primary complaint of erythema and nodules on the right side of her face for more than 1 year. Over a year ago, she developed a red rash on her right nasal alae after using a hot spring. She sought consultation at a nearby hospital and was initially diagnosed with “dermatitis” receiving topical treatment comprising fusidic acid, silicone oil cream, and tacrolimus ointment; however, the treatment proved ineffective, and the lesions progressively expanded. A skin biopsy was conducted at a different hospital. The pathological investigation demonstrated significant infiltration of plasma cells, histiocytes, and lymphocytes into the dermis. The histiocytes had complete lymphocytes, plasma cells, and neutrophils encapsulated within their cytoplasm, a condition referred to as “lymphocyte emperipolesis.” Immunohistochemistry showed positivity for S100 and CD68, scattered CD1a-positive cells, and a Ki-67 proliferation index of approximately 5%. A diagnosis of “RDD” was confirmed based on the clinical and pathological findings. The patient now requests additional diagnosis and treatment at our hospital. Dermatological assessment: the right side of the face and nasal ala had mildly infiltrated erythema, papules, and nodules varying in size from a wide bean to a coin. Several yellow nodules were centrally positioned on the right side of the face, with the potential for merging. The erythema had a somewhat annular configuration (Figure 1A). Since the onset of the disease, the patient’s mental condition, appetite, and sleep have been satisfactory, and bowel movements have been regular. The patient denied fever, night sweats, or weight loss. Physical examination revealed no palpable lymphadenopathy or hepatosplenomegaly. The laboratory findings revealed no instances of leukocytosis, elevated erythrocyte sedimentation rates, or polyclonal hypergammaglobulinemia. These results further substantiate the diagnosis of cutaneous-limited RDD (CRDD) over systemic involvement. The patient also had longstanding diabetes and hyperlipidemia, both of which were well-controlled on medication. The subject had no previous medical history of photosensitivity, oral ulcers, arthralgia, or drug or food allergies.

**FIGURE 2:**
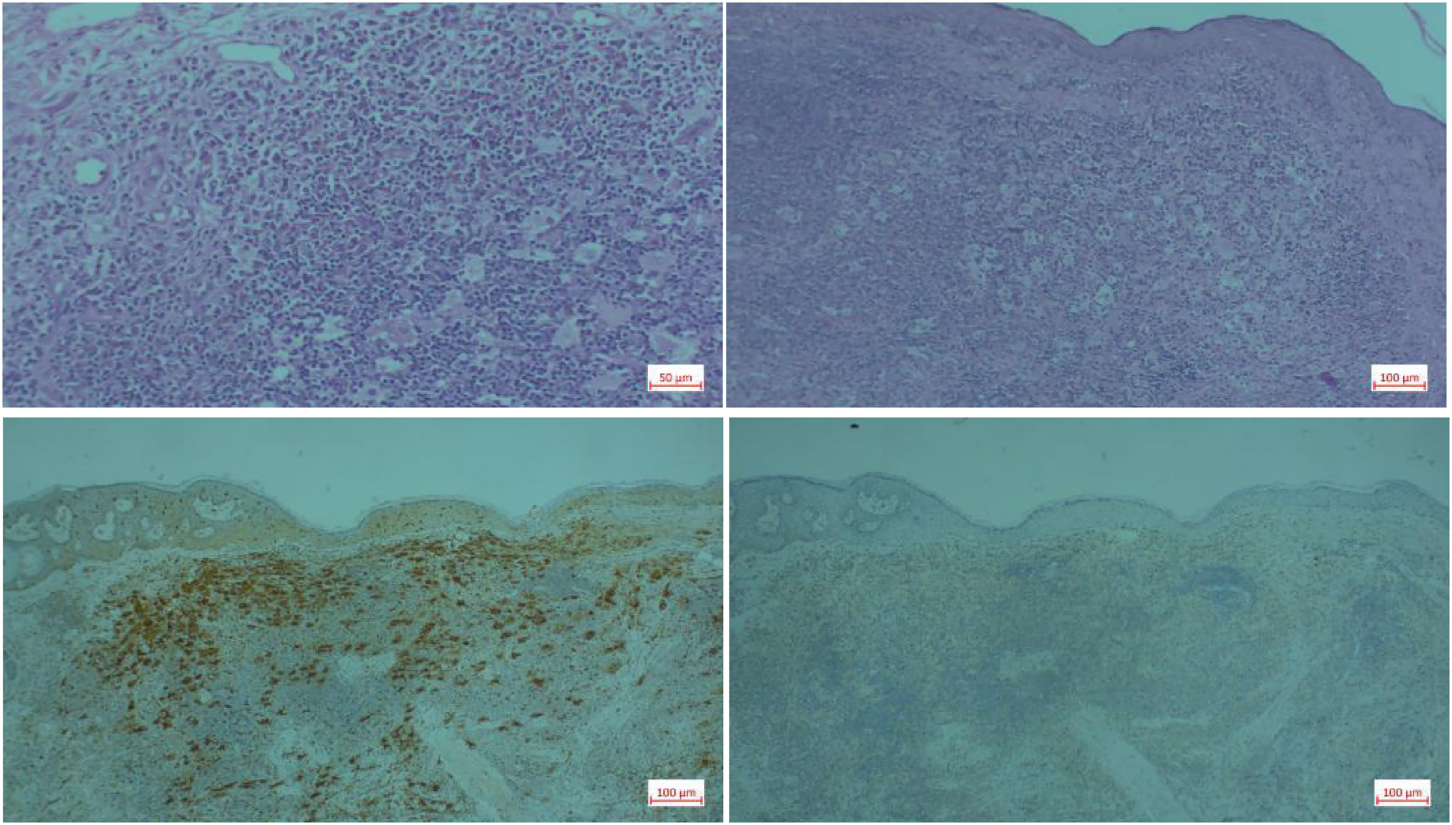
Histopathological results of the patient’s skin lesions.

Pertinent laboratory investigations: Autoantibody test results indicate a positive antinuclear antibody (1:100, cytoplasmic granular type) and a weakly positive mitochondrial M2 subtype, suggesting a potential underlying autoimmune background in the patient. The complete blood count, as well as liver and kidney function tests, were essentially normal. The patient had a remote history of tuberculous pleurisy, which was successfully treated and has remained quiescent. Prior to the initiation of JAK inhibitor therapy, baseline screening for latent tuberculosis infection was conducted, which revealed a positive T-SPOT.TB assay result. Chest CT imaging revealed mild chronic inflammatory changes that were consistent with a previous history of tuberculosis. No evidence of active tuberculosis was found (Table 1). Reflectance confocal microscopy revealed modest focal spongiosis in the stratum spinosum at the lesion site, along with dermal papillary and superficial vascular dilation, and various degrees of inflammatory cell infiltration surrounding the vessels. Skin histology revealed significant infiltration of plasma cells, histiocytes, and lymphocytes in the dermis, with intact lymphocytes, plasma cells, and neutrophils encapsulated within the cytoplasm of histiocytes, exemplifying the “lymphocyte emperipolesis phenomenon.” Immunohistochemical findings: S100 positive, CD68 positive, CD1a weakly positive, Ki67 (about 5%) (Figure 2). Considering the patient’s clinical presentation, skin pathology, and immunohistochemical findings, the diagnosis was established as sinus histiocytosis (RDD).

**TABLE 1:** Baseline laboratory findings prior to tofacitinib initiation.

Parameter	Result	Reference range	Notes
Antinuclear antibody (ANA)	Positive (1:100, cytoplasmic granular staining pattern)	Negative	Suggests underlying autoimmune background
T-SPOT.TB assay	Positive	Negative	Indicative of latent tuberculosis infection
Prothrombin time (PT)	11.8 s	11–14.2 s	–
Activated partial thromboplastin time (APTT)	28.3 s	28–43.5 s	–
White blood cell count (WBC)	5.94 × 10^9^/L	3.5–9.5 × 10^9^/L	–
Hemoglobin (HB)	139 g/L	115–150 g/L	–
Platelet count (PLT)	226 × 10^9^/L	125–350 × 10^9^/L	–
Neutrophil percentage	57.5%	40%–75%	–
Lymphocyte percentage	36.6%	20%–50%	–
Chest CT	Mild chronic infectious changes in both lungs	N/A	Consistent with positive T-SPOT; indicates prior tuberculosis infection

Treatment and clinical course: As the patient declined surgical intervention, systemic and local medical therapies were pursued instead. The initial treatment plan comprised oral thalidomide (75 mg once daily) and methotrexate (10 mg once weekly, alongside 5 mg of folic acid weekly). Following a suboptimal response after 2 weeks on this regimen, fire needle therapy was added to the ongoing oral medications with the patient’s informed consent. This therapy was administered once per week using local, scattered, and surrounding needling techniques. After an additional 2 weeks of this combined therapy (thalidomide, methotrexate, and fire needling), the papules and nodules showed modest flattening (Figure 1B). Given the limited improvement, a strategic shift was made. The thalidomide, methotrexate and fire needle treatments were discontinued and tofacitinib monotherapy (5 mg twice daily) was initiated. After 2 months of tofacitinib monotherapy, the erythematous nodules on the right cheek had significantly flattened and faded (Figure 1C). After 4 months of continuous tofacitinib treatment, the lesions had regressed further, becoming notably softer and flatter than at baseline (Figure 1D). The patient remains under follow-up. At the time of writing, she has completed 4 months of tofacitinib therapy, experiencing sustained improvement with no significant discomfort or adverse events reported.

## 3 Discussion

The management of refractory cutaneous RDD (CRDD) remains a clinical challenge, with no standardized therapeutic protocol. It is presently considered intimately associated with alterations in the MAPK/ERK signaling pathway and the deregulation of the local immunological inflammatory response (13). Despite the utilization of surgery, glucocorticoids, and conventional immunosuppressants for patients exhibiting pronounced symptoms or systemic involvement, a cohesive and effective therapeutic strategy for refractory CRDD remains elusive (3, 14, 15). A systematic review (involving 118 patients) demonstrated that surgical resection achieved the highest complete remission rate at 94%, whereas systemic glucocorticoids, methotrexate, thalidomide, and other frequently utilized treatments exhibited variable efficacy, with some patients encountering the risk of adverse reactions (16). In situations that are refractory, numerous, or cosmetically impacted, local physical therapies, including pulsed dye laser and photodynamic treatment, have demonstrated promising applications and aesthetic outcomes. The process may involve the regulation of the local immune microenvironment and the inhibition of aberrant tissue cell growth (17). This study presents the first successful case of treating facial CRDD utilizing the JAK inhibitor tofacitinib in conjunction with traditional Chinese medicine fire needling. At the 4-month follow-up, the patient’s skin lesions exhibited considerable improvement, and no apparent adverse reactions were seen, offering a novel strategy for the management of CRDD.

Concerning etiology, RDD is presently thought to be intricately linked to alterations in the MAPK/ERK signaling pathway and the deregulation of localized immunological inflammatory responses. In certain instances, it has been established that RDD propels disease progression and serves as the foundation for targeted therapy utilizing MEK inhibitors (such as trametinib and cobimetinib) (13, 18). While MEK inhibitors have demonstrated efficacy in RDD, particularly in cases with documented MAPK pathway mutations, their use was not pursued in this patient due to the absence of genetic confirmation and the favorable safety profile of tofacitinib. In consideration of the fact that the patient’s disease was confined to the skin without systemic involvement, the well-characterized safety and ease of administration of tofacitinib were prioritized over the class-specific toxicities associated with MEK inhibition. Histopathologically, RDD lesions are characterized by extensive histiocytic infiltration and the classic “lymphocyte emperipolesis,” demonstrating prominent inflammatory and immune cell infiltration. This suggests that immune-inflammatory mechanisms play a significant role in disease pathogenesis. Therefore, immunomodulation may serve as a key therapeutic target (4, 5). As a key signaling node, the JAK-STAT signaling pathway is the principal transduction mechanism for many cytokines, including IL-6, IFN-γ, and GM-CSF, and plays a significant role in regulating immune cell activation, differentiation, and inflammatory response (6). Tofacitinib, a highly selective JAK1/JAK3 inhibitor, effectively obstructs the signal transduction of downstream pro-inflammatory molecules by inhibiting this pathway, consequently modulating the aberrant activation of tissue cells and lymphocyte function and reforming the immune microenvironment (7, 8).

In recent years, JAK inhibitors have demonstrated significant efficacy in certain immune-mediated inflammatory dermatological conditions (9, 19–21). Upadacitinib has effectively treated patients with bullous pemphigoid complicated by psoriasis, illustrating its therapeutic promise in intricate immunological contexts (20). Tofacitinib has garnered clinical evidence for its efficacy in treating refractory autoimmune bullous illnesses and inflammatory granulomatous disorders (21). These studies offer theoretical validation and clinical guidance for the utilization of JAK inhibitors in RDD. The patient’s serum exhibited positivity for antinuclear antibodies and mitochondrial M2 subtype antibodies prior to treatment, signifying a systemic autoimmune background and reinforcing the theoretical justification for employing immunomodulatory therapy. The choice of tofacitinib for RDD treatment is grounded in both clinical experience and its molecular foundation. Tofacitinib may not only suppress the localized inflammatory response of RDD but also possess the capacity to modulate the systemic immunological dysregulation, effecting a strategic transition from “treating the localized disease” to “regulating overall immunity.” From a traditional Chinese medicine perspective, the lesions correspond to blood stasis and phlegm-dampness stagnation, for which fire needle therapy is indicated to warm meridians and resolve nodules (11, 12, 22). Interestingly, this traditional mechanism may converge with the modern concept that controlled physical stimulation (like fire needling) can modulate local immune responses and microcirculation (23), potentially aiding in lesion resolution. In this instance, we employed it as a supplementary local intervention to facilitate the regression of localized skin lesions. The specific mechanism requires additional investigation.

To the best of our knowledge, this is the first report documenting the use of tofacitinib, a JAK inhibitor, in the treatment of cutaneous Rosai-Dorfman disease, with prior adjunctive fire needle therapy. While fire needle therapy was associated with modest initial improvement, the most substantial and sustained regression of skin lesions occurred following the initiation of tofacitinib monotherapy. We hypothesize that these two modalities may target different aspects of the disease pathophysiology: fire needling may exert local immunomodulatory effects through thermal and micro-traumatic stimulation, potentially improving local circulation and disrupting the lesional microenvironment; tofacitinib, in contrast, provides systemic immune modulation by inhibiting JAK-STAT signaling, thereby dampening pathogenic cytokine cascades. Although administered sequentially in this case, their potentially complementary mechanisms warrant further investigation. This case suggests that for patients with CRDD refractory to conventional immunosuppressants, a sequential approach involving initial local therapy followed by systemic JAK inhibition may offer a promising therapeutic strategy. However, this hypothesis requires validation in prospective studies with larger cohorts and standardized treatment protocols.

## 4 Limitations

This report is subject to several limitations inherent to its single retrospective case design. First, the observed clinical improvement must be interpreted with caution, as the specific therapeutic contribution of tofacitinib cannot be definitively distinguished from that of prior interventions, including thalidomide, methotrexate, and fire needle therapy. Second, the 4-month follow-up period is too short to assess long-term efficacy, potential relapse, or the safety profile of sustained JAK inhibition. Third, the absence of correlative biomarker data—such as MAPK pathway mutation analysis or serial quantification of inflammatory cytokines and phospho-STAT signaling in tissue—limits mechanistic insight and precludes objective confirmation of target engagement. Fourth, due to financial constraints, the patient opted to undergo follow-up laboratory testing (including liver function tests, blood glucose, and abdominal ultrasound) at a local facility and was subsequently lost to follow-up; consequently, objective safety data beyond clinical observation are unavailable. Finally, while fire needling is rooted in traditional practice, its use in Rosai-Dorfman disease lacks standardized protocols and biological validation, rendering its role in the observed outcome speculative. These constraints underscore the preliminary nature of our findings and highlight the need for prospective, controlled studies with integrated translational research to validate this therapeutic approach.

## Data Availability

The original contributions presented in this study are included in this article/supplementary material, further inquiries can be directed to the corresponding author.

## References

[1] McClain KL Bigenwald C Collin M Haroche J Marsh RA Merad M et al. Histiocytic disorders. *Nat Rev Dis Primers*. (2021) 7:73. 10.1038/s41572-021-00307-9 34620874 PMC10031765

[2] Sampaio R Silva L Catorze G Viana I. Cutaneous Rosai-Dorfman disease: a challenging diagnosis. *BMJ Case Rep*. (2021) 14:e239244. 10.1136/bcr-2020-239244 33541998 PMC7868278

[3] Gawdzik A Ziarkiewicz-Wróblewska B Chlebicka I Jankowska-Konsur A Szepietowski JC Maj J. Cutaneous Rosai-Dorfman disease: a treatment challenge. *Dermatol Ther*. (2021) 11:1443–8. 10.1007/s13555-021-00557-1 34143402 PMC8322247

[4] Chen LYC Slack GW Carruthers MN. IgG4-related disease and Rosai-Dorfman-Destombes disease. *Lancet*. (2021) 398:1213–4. 10.1016/S0140-6736(21)01812-2 34600619

[5] Goyal G Ravindran A Young JR Shah MV Bennani NN Patnaik MM et al. Clinicopathological features, treatment approaches, and outcomes in Rosai-Dorfman disease. *Haematologica*. (2020) 105:348–57. 10.3324/haematol.2019.219626 31004029 PMC7012468

[6] Fortelny N Farlik M Fife V Gorki AD Lassnig C Maurer B et al. JAK-STAT signaling maintains homeostasis in T cells and macrophages. *Nat Immunol*. (2024) 25:847–59. 10.1038/s41590-024-01804-1 38658806 PMC11065702

[7] Nordmann TM Anderton H Hasegawa A Schweizer L Zhang P Stadler PC et al. Spatial proteomics identifies JAKi as treatment for a lethal skin disease. *Nature*. (2024) 635:1001–9. 10.1038/s41586-024-08061-0 39415009 PMC11602713

[8] Ruperto N Brunner HI Synoverska O Ting TV Mendoza CA Spindler A et al. Tofacitinib in juvenile idiopathic arthritis: a double-blind, placebo-controlled, withdrawal phase 3 randomised trial. *Lancet*. (2021) 398:1984–96. 10.1016/S0140-6736(21)01255-1 34767764

[9] Chovatiya R Paller AS. JAK inhibitors in the treatment of atopic dermatitis. *J Allergy Clin Immunol*. (2021) 148:927–40. 10.1016/j.jaci.2021.08.009 34437922 PMC10166130

[10] Virtanen A Spinelli FR Telliez JB O’Shea JJ Silvennoinen O Gadina M. JAK inhibitor selectivity: new opportunities, better drugs? *Nat Rev Rheumatol*. (2024) 20:649–65. 10.1038/s41584-024-01153-1 39251770

[11] Du L Cao Z Wei J Li M Han C Zhang C. Fire needle pretreatment with 5-aminolevulinic acid photodynamic therapy combined with low-dose isotretinoin in the treatment of severe refractory nodulocystic acne. *Photodiagnosis Photodyn Ther*. (2024) 47:104215. 10.1016/j.pdpdt.2024.104215 38735352

[12] Tang L Huang F Zhou X Zhao M Huang M. Fire needle combined with photodynamic therapy for cutaneous infectious granulomatosis caused by mycobacterium chelonae: a case report. *Photodiagnosis Photodyn Ther*. (2024) 45:103836. 10.1016/j.pdpdt.2023.103836 37813272

[13] Abeykoon JP Rech KL Young JR Ravindran A Ruan GJ Dasari S et al. Outcomes after treatment with cobimetinib in patients with rosai-dorfman disease based on KRAS and MEK Alteration Status. *JAMA Oncol*. (2022) 8:1816–20. 10.1001/jamaoncol.2022.4432 36201194 PMC9539729

[14] Bruce-Brand C Schneider JW Schubert P. Rosai-Dorfman disease: an overview. *J Clin Pathol*. (2020) 73:697–705. 10.1136/jclinpath-2020-206733 32591351

[15] Ngo TQ Dorwal P Oh D. Successful treatment of Rosai-Dorfman disease in the era of targeted therapy. *Intern Med J*. (2025) 55:1042–4. 10.1111/imj.70108 40454972

[16] Dhrif O Litaiem N Lahmar W Fatnassi F Slouma M Zeglaoui F. Cutaneous Rosai-Dorfman disease: a systematic review and reappraisal of its treatment and prognosis. *Arch Dermatol Res*. (2024) 316:393. 10.1007/s00403-024-02982-6 38878198

[17] Wang QX Luo SY Zhou KY Fang S. Facial cutaneous Rosai-Dorfman disease treated with pulsed dye laser: a case report and literature review. *An Bras Dermatol*. (2025) 100:204–7. 10.1016/j.abd.2024.05.002 39521706 PMC11745284

[18] Diamond EL Durham BH Ulaner GA Drill E Buthorn J Ki M et al. Efficacy of MEK inhibition in patients with histiocytic neoplasms. *Nature*. (2019) 567:521–4. 10.1038/s41586-019-1012-y 30867592 PMC6438729

[19] Ingrassia JP Maqsood MH Gelfand JM Weber BN Bangalore S Lo Sicco KI et al. Cardiovascular and venous thromboembolic risk with JAK inhibitors in immune-mediated inflammatory skin diseases: a systematic review and meta-analysis. *JAMA Dermatol*. (2024) 160:28–36. 10.1001/jamadermatol.2023.4090 37910098 PMC10620674

[20] Su F Wang T Qin Q Xie Z. Upadacitinib for the management of bullous pemphigoid coexisting with psoriasis vulgaris: a case report and literature review. *J Dermatolog Treat*. (2024) 35:2302394. 10.1080/09546634.2024.2302394 38263708

[21] Moriana C Moulinet T Jaussaud R Decker P. JAK inhibitors and systemic sclerosis: a systematic review of the literature. *Autoimmun Rev*. (2022) 21:103168. 10.1016/j.autrev.2022.103168 35944611

[22] Su Y Xu Q Zhang C Zhang C. Photodynamic therapy pre-treated by fire needle combined with isotretinoin in the treatment of refractory perifolliculitis capitis abscedens et suffodiens: case report. *Photodiagnosis Photodyn Ther*. (2021) 33:102103. 10.1016/j.pdpdt.2020.102103 33359162

[23] Oh JE Kim SN. Anti-inflammatory effects of acupuncture at ST36 point: a literature review in animal studies. *Front Immunol*. (2022) 12:813748. 10.3389/fimmu.2021.813748 35095910 PMC8790576

